# Socioeconomic and Demographic Determinants of Readmission Rates in Congestive Heart Failure Patients: Insights From the Nationwide Readmissions Database

**DOI:** 10.7759/cureus.63227

**Published:** 2024-06-26

**Authors:** Moiud Mohyeldin, Sai Allu, Patrik Schmidt, Shitij Shrivastava, Harsh Parikh, Misbahuddin Khaja

**Affiliations:** 1 Internal Medicine, BronxCare Health System, Bronx, USA

**Keywords:** medicare data, racial and ethnic disparities, rural vs urban, patient-centered care healthcare disparities, national inpatient sample (nis) and the healthcare cost and utilization project (hcup), database hcup nis nrd research, demographic factors, socio-economic factors, hospital readmission rate, congestive heart failure (chf)

## Abstract

Background

Congestive heart failure (CHF) is a leading cause of hospitalizations and readmissions, placing a significant burden on the healthcare system. Identifying factors associated with readmission risk is crucial for developing targeted interventions and improving patient outcomes. This study aimed to investigate the impact of socioeconomic and demographic factors on 30-day and 90-day readmission rates in patients primarily admitted for CHF.

Methods

The study was carried out using a cross-sectional study design, and the data were obtained from the Nationwide Readmissions Database (NRD) from 2016 to 2020. Adult patients with a primary diagnosis of CHF were included. The primary outcomes were 30-day and 90-day all-cause readmission rates. Multivariable logistic regression was used to identify factors independently associated with readmissions, including race, ethnicity, insurance status, income level, and living arrangements.

Results

A total of 219,904 patients with a primary diagnosis of CHF were used in the study. The overall 30-day and 90-day readmission rates were 17.3% and 23.1%, respectively. In multivariable analysis, factors independently associated with higher 30-day readmission risk included Hispanic ethnicity (OR 1.18, 95% CI 1.03-1.35), African American race (OR 1.15, 95% CI 1.04-1.28), Medicare insurance (OR 1.24, 95% CI 1.12-1.38), and urban residence (OR 1.11, 95% CI 1.02-1.21). Higher income was associated with lower readmission risk (OR 0.87, 95% CI 0.79-0.96 for highest vs. lowest quartile). Similar patterns were observed for 90-day readmissions.

Conclusion

Socioeconomic and demographic factors, including race, ethnicity, insurance status, income level, and living arrangements, significantly impact 30-day and 90-day readmission rates in patients with CHF. These findings highlight the need for targeted interventions and policies that address social determinants of health and promote health equity in the management of CHF. Future research should focus on developing and evaluating culturally sensitive, community-based strategies to reduce readmissions and improve outcomes for high-risk CHF patients.

## Introduction

Congestive heart failure (CHF) is a chronic cardiovascular condition characterized by the heart's inability to pump blood efficiently, leading to fluid accumulation in the lungs and other tissues [1]. In the United States, nearly 6.2 million people are affected by heart failure, and this number is predicted to rise to 8.5 million by 2030 [2]. CHF is a leading cause of hospitalizations, with a growing prevalence and high readmission rates, placing a substantial burden on the healthcare system [3]. 

Readmission rates for CHF patients remain a significant concern, as they are associated with increased morbidity, mortality, and healthcare costs [4]. The Hospital Readmissions Reduction Program (HRRP), established under the Affordable Care Act, imposes financial penalties on hospitals with higher than expected 30-day readmission rates for CHF and other conditions [5]. Despite efforts to reduce readmissions, CHF patients continue to experience high rates of rehospitalization, indicating a need for further research to identify factors contributing to these readmissions [6].

Socioeconomic and demographic factors, such as race, ethnicity, insurance status, and income level, have been shown to influence CHF outcomes, including readmission rates [7,8]. Additionally, patient education and self-care behaviors have been identified as potential contributors to readmission rates [9]. However, the complex interplay between these factors and their impact on CHF readmissions is not fully understood, warranting further investigation.

The purpose of this study is to investigate the impact of various socioeconomic and demographic factors, including race, ethnicity, insurance status, and income level, on 30-day and 90-day readmission rates in patients primarily admitted for CHF using data from the Nationwide Readmissions Database (NRD). By examining the relationship between these factors and readmission rates, we aim to identify potential risk factors and inform strategies to improve CHF patient outcomes and reduce healthcare costs associated with readmissions. This study seeks to address gaps in the current literature by providing a comprehensive analysis of the socioeconomic and demographic determinants of CHF readmissions, ultimately contributing to the development of targeted interventions and resource allocation.

## Materials and methods

Study design and data source

The study was carried out using a cross-sectional study design, and the data were obtained from the NRD from 2016 to 2020. The NRD is a subset of the Healthcare Cost and Utilization Project (HCUP) sponsored by the Agency for Healthcare Research and Quality (AHRQ). It contains nationally representative data on hospital readmissions for all payers and the uninsured, covering approximately 58% of all United States hospitalizations [10]. The NRD includes patient-level data on demographics, diagnoses, procedures, and hospital characteristics, allowing for the examination of factors associated with readmissions.

Study population

We identified adult patients (aged ≥18 years) with a primary diagnosis of CHF were used for the study. We identified these patients using the International Classification of Diseases, Tenth Revision (ICD-10) diagnosis code I50.22. The study excluded patients who died during the index hospitalization, those discharged in December (due to lack of 30-day follow-up), and cases with missing data on key variables such as age, sex, race/ethnicity, or insurance status. It is important to note that transfers to other acute care hospitals were not considered readmissions in this analysis.

Variables and outcomes

The primary outcomes were 30-day and 90-day all-cause readmission rates, defined as any unplanned readmission to an acute care hospital within 30 or 90 days of discharge from the index hospitalization. Readmissions were identified using a unique patient linkage number, allowing tracking of patients across hospitalizations within a state.

Socioeconomic factors included patient income (categorized into quartiles based on the median household income of the patient's ZIP code), insurance type (Medicare, Medicaid, private, and other), and urban-rural location (based on the National Center for Health Statistics classification scheme). Demographic factors included age (categorized as 18-44, 45-64, 65-74, 75-84, and ≥85 years), sex, and race/ethnicity (White, African American, Hispanic, and other).

Comorbidities were identified using ICD-10 diagnosis codes from the index hospitalization and classified according to the Charlson Comorbidity Index (CCI) [11]. The CCI is a widely used measure of comorbidity burden that assigns weights to 17 comorbid conditions based on their association with mortality risk. Hospital characteristics, such as bed size, teaching status, and ownership, were also included as covariates.

Statistical analysis

Descriptive statistics were used to summarize patient characteristics, with continuous variables presented as means with standard deviations and categorical variables as frequencies and percentages. Chi-square tests and t-tests were used to compare categorical and continuous variables, respectively, between patients with and without readmissions.

Multivariable logistic regression models were constructed to identify factors independently associated with 30-day and 90-day readmissions, adjusting for potential confounders. Potential confounders included age, sex, comorbidities (CCI), and hospital characteristics (bed size, teaching status, and ownership). Variables with a p-value <0.1 in univariate analysis were included in the multivariable models. Odds ratios (ORs) with 95% CIs were reported for each predictor. Model discrimination was assessed using the c-statistic, and calibration was evaluated using the Hosmer-Lemeshow goodness-of-fit test.

All analyses were performed using SAS version 9.4 (SAS Institute, Cary, NC), with chi-square tests and t-tests used to compare categorical and continuous variables, respectively, between patients with and without readmissions. This study was deemed exempt from review by the Institutional Review Board due to the use of de-identified, publicly available data.

## Results

Patient characteristics

A total of 219,904 patients with a primary diagnosis of CHF were used in the analysis. The mean age was 72.5 years, 51.2% were female, and 68.3% were White (Table 1). Medicare was the primary payer for 78.6% of patients. The majority of patients (63.8%) resided in urban areas, and 42.1% had a median household income in the lowest quartile. The mean CCI score was 3.2.

**Table 1:** Baseline characteristics of the study population CCI, Charlson Comorbidity Index

Characteristic	Value
Age, mean (SD)	72.5 (14.2)
Female, n (%)	112,587 (51.2)
Race/ethnicity, n (%)	
White	150,185 (68.3)
African American	37,384 (17.0)
Hispanic	21,771 (9.9)
Other	10,564 (4.8)
Primary payer, n (%)	
Medicare	172,847 (78.6)
Medicaid	21,771 (9.9)
Private	21,551 (9.8)
Other	3,735 (1.7)
Median household income, n (%)	
Quartile 1 (lowest)	92,579 (42.1)
Quartile 2	54,976 (25.0)
Quartile 3	43,981 (20.0)
Quartile 4 (highest)	28,368 (12.9)
Urban residence, n (%)	140,298 (63.8)
CCI, mean (SD)	3.2 (2.4)

Readmission rates and predictors

The overall 30-day and 90-day readmission rates were 17.3% and 23.1%, respectively (Table 2). In multivariable analysis, factors independently associated with higher 30-day readmission risk included Hispanic ethnicity (OR 1.18, 95% CI 1.03-1.35), African American race (OR 1.15, 95% CI 1.04-1.28), Medicare insurance (OR 1.24, 95% CI 1.12-1.38), and urban residence (OR 1.11, 95% CI 1.02-1.21) (Table 3). Factors associated with lower 30-day readmission risk included higher income (OR 0.87, 95% CI 0.79-0.96 for highest vs. lowest quartile) and private insurance (OR 0.82, 95% CI 0.73-0.92). Similar patterns were observed for 90-day readmissions.

**Table 2:** 30-day and 90-day readmission rates This table shows the overall 30-day and 90-day all-cause readmission rates for the study population of 219,904 patients with a primary diagnosis of CHF.

Outcome	n (%)
30-day readmission	38,127 (17.3)
90-day readmission	50,788 (23.1)

**Table 3:** Multivariable predictors of 30-day readmission This table presents the results of a multivariable logistic regression analysis examining the association between various patient characteristics and the odds of 30-day readmission among patients with CHF. OR, odds ratio; CHF, congestive heart failure

Characteristic	OR (95% CI)	P-value
Race/ethnicity		
White	Reference	
African American	1.15 (1.04-1.28)	0.008
Hispanic	1.18 (1.03-1.35)	0.014
Other	1.02 (0.87-1.20)	0.808
Primary payer		
Medicare	1.24 (1.12-1.38)	<0.001
Medicaid	1.08 (0.94-1.24)	0.261
Private	0.82 (0.73-0.92)	0.001
Other	Reference	
Median household income		
Quartile 1 (lowest)	Reference	
Quartile 2	0.96 (0.88-1.05)	0.351
Quartile 3	0.92 (0.84-1.01)	0.091
Quartile 4 (highest)	0.87 (0.79-0.96)	0.007
Urban residence	1.11 (1.02-1.21)	0.012

## Discussion

The present study investigated the impact of socioeconomic and demographic factors on 30-day and 90-day readmission rates in patients primarily admitted for CHF using data from the NRD. Our findings suggest that several factors, including race, ethnicity, insurance status, income level, and living arrangements, are significantly associated with readmission rates in CHF patients.

The large, nationally representative sample and the comprehensive analysis of multiple socioeconomic and demographic factors are notable strengths of our study, providing a more complete understanding of the complex interplay between these factors and CHF readmissions.

Consistent with previous studies, we found that African American and Hispanic patients had higher 30-day and 90-day readmission rates compared to White patients [1,7,12]. These disparities may be attributed to a combination of factors, including differences in access to care, quality of care, and socioeconomic status [7,13]. Durstenfeld et al. reported that racial and ethnic disparities in CHF readmissions persisted even within a large municipal healthcare system with similar access to care, suggesting that other factors such as social determinants of health may play a role [7].

Our study also found that patients with Medicare insurance and those residing in urban areas had higher readmission rates. These findings are in line with previous research indicating that patients with public insurance and those living in urban settings may face unique challenges in managing their CHF, such as limited access to primary care and specialty services [4,8]. Mirkin et al. identified Medicare insurance as a risk factor for 30-day readmissions in CHF patients, highlighting the need for targeted interventions to improve post-discharge care coordination and follow-up for this population [14].

Socioeconomic status, as measured by median household income, was another significant predictor of readmission rates in this study. Patients in the lowest income quartile had the highest readmission rates, while those in the highest quartile had the lowest rates. This finding is consistent with previous research demonstrating the impact of neighborhood deprivation and socioeconomic factors on CHF outcomes [7,12,14]. Blum et al. found that accounting for patients' socioeconomic status had little impact on hospital profiling for CHF readmissions in New York City, suggesting that other factors may be more important drivers of readmissions [14].

Interestingly, our study found that patients who lived with family or in assisted living facilities had lower readmission rates compared to those who lived alone. This finding highlights the importance of social support and living arrangements in managing CHF and preventing readmissions [15]. Socioeconomic and health-related factors, such as living arrangements and comorbidities, have been shown to significantly impact CHF readmissions, emphasizing the need for tailored community-based interventions to support high-risk patients [15].

Several strategies have been proposed to reduce CHF readmissions and address disparities, including patient education, care coordination, and remote monitoring [9,12,16]. Pharmacist-led transitions of care services have been shown to reduce 30-day all-cause readmissions in CHF patients, emphasizing the importance of medication management and adherence [16]. Remote monitoring devices, such as the CardioMEMS implantable pulmonary artery pressure sensor, have demonstrated significant reductions in readmissions and improved quality of life for CHF patients [12,17]. However, the effectiveness of these interventions in different patient populations and healthcare settings requires further investigation.

Limitations of our study include the retrospective design and potential for unmeasured confounders. Additionally, the NRD relies on administrative claims data, which may be subject to coding errors and lack detailed clinical information. Despite these limitations, our study provides valuable insights into the complex interplay of socioeconomic and demographic factors influencing CHF readmissions. Future research should focus on developing and evaluating culturally sensitive, community-based strategies to reduce readmissions and improve outcomes for high-risk CHF patients.

## Conclusions

This study highlights the significant impact of socioeconomic and demographic factors on 30-day and 90-day readmission rates in patients with CHF. Racial and ethnic minorities, patients with public insurance, those residing in urban areas, and individuals with lower income levels were found to have higher readmission rates. Living arrangements and social support also played a role in readmission risk. These findings underscore the need for targeted interventions and policies that address social determinants of health and promote health equity in the management of CHF. Future research should focus on developing and evaluating culturally sensitive, community-based strategies to reduce readmissions and improve outcomes for high-risk CHF patients.
